# The public health significance of finding autochthonous melioidosis cases in the continental United States

**DOI:** 10.1371/journal.pntd.0011550

**Published:** 2023-08-24

**Authors:** Alfredo G. Torres

**Affiliations:** Department of Microbiology and Immunology; Department of Pathology, Sealy Center for Vaccine Development; University of Texas Medical Branch, Galveston, Texas, United States of America; Yale University School of Medicine, UNITED STATES

## Abstract

Recently, the pathogen that causes melioidosis, *Burkholderia pseudomallei*, was found in the Gulf Coast region of Mississippi, United States of America, associated with human cases and as bacteria in the soil of affected areas. Therefore, the Centers for Disease Control and Prevention has declared the pathogen as endemic in the continental United States for the first time. This viewpoint discusses some issues that the research, public health communities, and government agencies need to address.

Melioidosis is an infectious disease caused by *Burkholderia pseudomallei*, a bacterial pathogen that can infect humans and other animals [1]. Human cases of the disease have been thought to be restricted to Southeast Asia and Northern Australia, where the pathogen is endemic and found in the soil and water [2]. However, in recent years, the presence of *B*. *pseudomallei* has been demonstrated in the African and American continents and in the Caribbean islands as well as the rest of Asia [3–6]. The continental United States had never confirmed an endemic case of the disease and it was presumed that all the melioidosis patients from the continental US acquired the disease while traveling to endemic areas around the globe [7,8]. In fact, melioidosis was considered a traveler’s disease because incidence in the continental US was associated with international acquisition [9]. However, this changed recently when the Centers for Disease Control and Prevention (CDC) reported 2 cases of melioidosis in Mississippi residents occurring in 2020 and 2022 [10]. These cases were unique in that they were unrelated, but the 2 patients lived near each other in the Gulf Coast region of southern Mississippi. Environmental samples collected from soil and water were positive for *B*. *pseudomallei* and showed genetic similarity to the human clinical isolates, suggesting that the local environmental exposure were the source of infection [11]. Even though all human cases are independently acquired from the environment, in January of this year, a third case of melioidosis was identified in the same county as the other 2 cases and the clinical samples showed genetic similarity to the previous cases, providing further evidence of locally acquired infections [12].

Based on these findings, the Gulf Coast region of Mississippi has been declared by the CDC as an endemic area. Further investigation is needed to better understand the public health implications of these cases. Here are some key issues the research and public health communities, as well as the government agencies, need to consider as areas of concern:

1. Emerging infectious disease: Melioidosis is considered an emerging infectious disease in regions outside the well-established endemic areas, but still not considered a neglected tropical disease [13], so detection in the environment of the continental US suggests a potential expansion in the geographic range of the *B*. *pseudomallei*. Establishment of the disease in this environment that favors the survival of the pathogen should be seriously considered as a public health threat for the residents of the Gulf coast region [8].
2. Disease awareness and diagnosis: In regions where the disease is/was not endemic, like the continental US, physicians are not familiar with disease symptomatology thus melioidosis may be given little consideration during initial diagnoses. Further, automated systems that perform bacterial identification may misidentify *B*. *pseudomallei* [14]. Therefore, accurate identification of melioidosis cases should be urged among healthcare providers, including the use of culture methods which are still considered the gold standard for the diagnosis of melioidosis which provides conclusive evidence for the presence of the pathogen.
3. Public health preparedness: The identification of melioidosis cases highlights the need for federal and state public health agencies to be prepared for the emergence of additional cases of the disease. This includes surveillance systems to detect and monitor cases, laboratory capacity and training for accurate diagnosis, and response protocols to prevent further spread. Identifying and investigating melioidosis cases in the continental US can serve as a wake-up call to strengthen public health preparedness efforts for this and other emerging infectious diseases.
4. Travel and global health: The identification of melioidosis cases in the US Gulf coast region may have implications for global health. It is highlighting the importance of adding melioidosis as a disease that can potentially be acquired by travelers to this region. Additionally, it underscores the need for collaboration among US states in monitoring and responding to unexpected melioidosis cases, particularly from those local individuals that have never traveled outside that area to another endemic region.
5. Need of financial support for diagnosis, research, and therapies: Because melioidosis is not included in the list of neglected tropical diseases and thought to affect few countries, the investment in research and development has been minimum. The potential use of the pathogen as a biothreat agent invigorated the financial support to identify vaccine candidates and therapeutics for some years. However, this funding has not continued, making investigation of this pathogen even a more of a challenge, due to the complex and regulated laboratory environment to work with a CDC select agent, requiring specialized biocontainment facilities. With the appearance of the disease in the continental US and other non-endemic regions, it is evident that financial support is needed to develop more accurate detection methods, characterize those clinical isolates that represent novel clones of *B*. *pseudomallei*, further develop therapeutic approaches to combat the disease, and perform surveillance to determine whether the pathogen has spread to other non-endemic regions. This information is crucial for implementing appropriate infection control measures in case a wider spread is demonstrated.

In summary, finding human cases of melioidosis in the Gulf Coast region of Mississippi has public health significance as it raises awareness about an emerging infectious disease, argues in favor of improving disease diagnosis, highlights the need for preparedness, emphasizes the importance of travel health and global health collaboration, and informs funding agencies of the need to support research and development in neglected areas of public health. These recommendations should also be considered by national and international health agencies to further improve public health measures to protect at-risk individuals.
